# Atypical Presentation of Creutzfeldt-Jacob Disease (CJD): Early Incontinence and Delayed Imaging in a Rapidly Progressive Dementia

**DOI:** 10.7759/cureus.97039

**Published:** 2025-11-17

**Authors:** Juman Baban, Aieman Saeed, Kyaw Thura, Hnin Thida Nwe, Crina Diaconu

**Affiliations:** 1 Internal Medicine, West Middlesex University Hospital, Isleworth, GBR; 2 Acute Medicine, West Middlesex University Hospital, Isleworth, GBR; 3 Neurology, West Middlesex University Hospital, Isleworth, GBR

**Keywords:** cjd, cognitive impairment, creutzfeldt-jacob disease, dementia, neuropsychiatric disease

## Abstract

Creutzfeldt-Jacob disease (CJD) is a rare, fatal prion disease that can present with neuropsychiatric features such as behavioral changes, visual and auditory hallucinations, and cognitive impairment. It follows a fulminant course with limited survival after symptom onset, which contributes to the limited number of diagnosed and reported cases. We report a 73-year-old woman, previously healthy and active, who exercised at the gym four days weekly, presenting with dizziness and impaired balance, followed by abrupt cognitive deterioration. Urinary incontinence developed a few weeks after cognitive decline, notably preceding the terminal stage of the illness. Magnetic resonance imaging (MRI) of the brain, which was initially deferred because of preexisting metalwork, revealed an abnormal fluid-attenuated inversion recovery (FLAIR) signal associated with reduced diffusivity involving the bilateral caudate, ventral putamen, thalami, and hippocampi symmetrically.

## Introduction

Creutzfeldt-Jakob disease (CJD) is a rare prion disorder with an incidence of approximately one case per million annually [1] and occurs in four forms: sporadic (85%), genetic (10-15%), iatrogenic, and variant [2]. Sporadic CJD (sCJD), which is the most common form, mostly presents later in life with a mean age of 67 years [3] and presents with rapidly progressive cognitive decline, myoclonus, and cerebellar dysfunction [4]. Rapidly progressive cognitive decline in elderly patients presents significant diagnostic challenges, particularly in distinguishing reversible conditions from invariably fatal neurodegenerative diseases such as CJD [5].

Diagnostic complexity increases when cerebellar symptoms arise in patients with active malignancy, as paraneoplastic syndromes such as cerebellar degeneration may mimic CJD [6]. We report a 73-year-old woman with breast cancer who developed rapidly progressive neurological decline over the course of eight weeks, initially manifesting as vertigo and ataxia. Soon after, she developed new urinary incontinence. Early manifestations of sCJD are often non-specific, like vertigo, fatigue, and sleep disturbance [6]. In this case, urinary incontinence appeared unusually early in the illness course, representing an atypical early manifestation [7,8]. This case highlights the diagnostic challenges posed by overlapping malignancy-related neurological symptoms and imaging limitations, with the final diagnosis established clinically and radiologically in the absence of real-time quaking-induced conversion (RT-QuIC) testing.

## Case presentation

We report a 73-year-old woman, previously robust and physically active, who presented with an eight-week history of worsening unsteadiness on her feet and dizziness. Three weeks after the dizziness onset, she developed deteriorating gait instability requiring the use of walking aids. This was completely unusual for her, as she had been previously healthy and regularly attended the gym several times a week. Around five weeks after the initial symptom, she developed a sudden decline in her cognitive state with disorientation and gradual memory impairment, leaving her dependent on her sister for all daily activities. The family became increasingly concerned during a holiday in Greece, where they observed that she had become increasingly forgetful and often confused. She was repeatedly asking her sister whether the flights had been rebooked. Notably, during the early progression of the disease, she was also found to have urinary incontinence, an uncommon early finding for prion disease, which reportedly may have begun earlier before the family first recognized it. No seizure, flu-like illness, or hallucinations were recognized by the family. She sought medical attention multiple times and underwent several computed tomography (CT) scans, all of which were reported to be normal. 

Her previous medical records included early breast cancer (on letrozole), seasonal asthma, and previous ear surgery with metallic staples. She was a lifelong non-smoker and didn't drink alcohol. 

Clinical examination on admission revealed disorientation to time, place, and person. She had nystagmus but no facial weakness. She demonstrated cerebellar signs, including impaired coordination, balance problems, and ataxia, which started peripherally and then axially, affecting all four limbs with truncal instability. In addition, she developed pyramidal signs such as upper limb tremor, generalized hyperreflexia, and a positive Hoffmann's sign with bilateral extensor plantar responses. Later on, she developed clonus as well. Extrapyramidal signs included cogwheel rigidity, bradykinesia, hypomimia, hypophonia, which later progressed to akinesia in all limbs, and myoclonus gradually involving all limbs. Weakness was noted proximally more than distally. She initially presented with expressive dysphasia and stuttering speech, which later progressed to intermittent mutism during admission.

Biochemical analysis revealed neutrophilic leucocytosis with persistently raised C-reactive protein (CRP). Cerebrospinal fluid (CSF) studies showed a mildly raised protein level (0.58 g/L; reference range: 0.15-0.45 g/L) and elevated albumin (470 mg/L; reference range: 0-320 mg/L). Examinations of flow cytometry, oligoclonal bands, immunoglobulin G, and microbial infection in the CSF showed no abnormalities. This helped to exclude inflammatory and infectious causes such as meningoencephalitis.

CSF viral polymerase chain reaction (PCR) panel (for enterovirus, herpes simplex viruses 1 and 2, varicella-zoster virus, cytomegalovirus, Epstein-Barr virus, adenovirus, parechovirus, and human herpesvirus 6) was entirely negative, ruling out viral meningoencephalitis. Neuronal antibody profile (serum) showed negative results for Hu, Yo, Ri, Ma2, CV2, and amphiphysin antibodies. Likewise, the neuronal immunofluorescence screen (Hu/ANNA-1, Yo/PCA-1, Ri/ANNA-2, and GAD antibodies) was negative, excluding paraneoplastic and autoimmune encephalitis. Autoimmune myositis antibody panel (including Ro-52, Jo-1, PL-7, PL-12, Mi-2, SRP, TIF1γ, MDA5, NXP2, and SAE1 antibodies) was also negative, which helped to exclude inflammatory or connective tissue disease-related neurological syndromes. 

**Figure 1:**
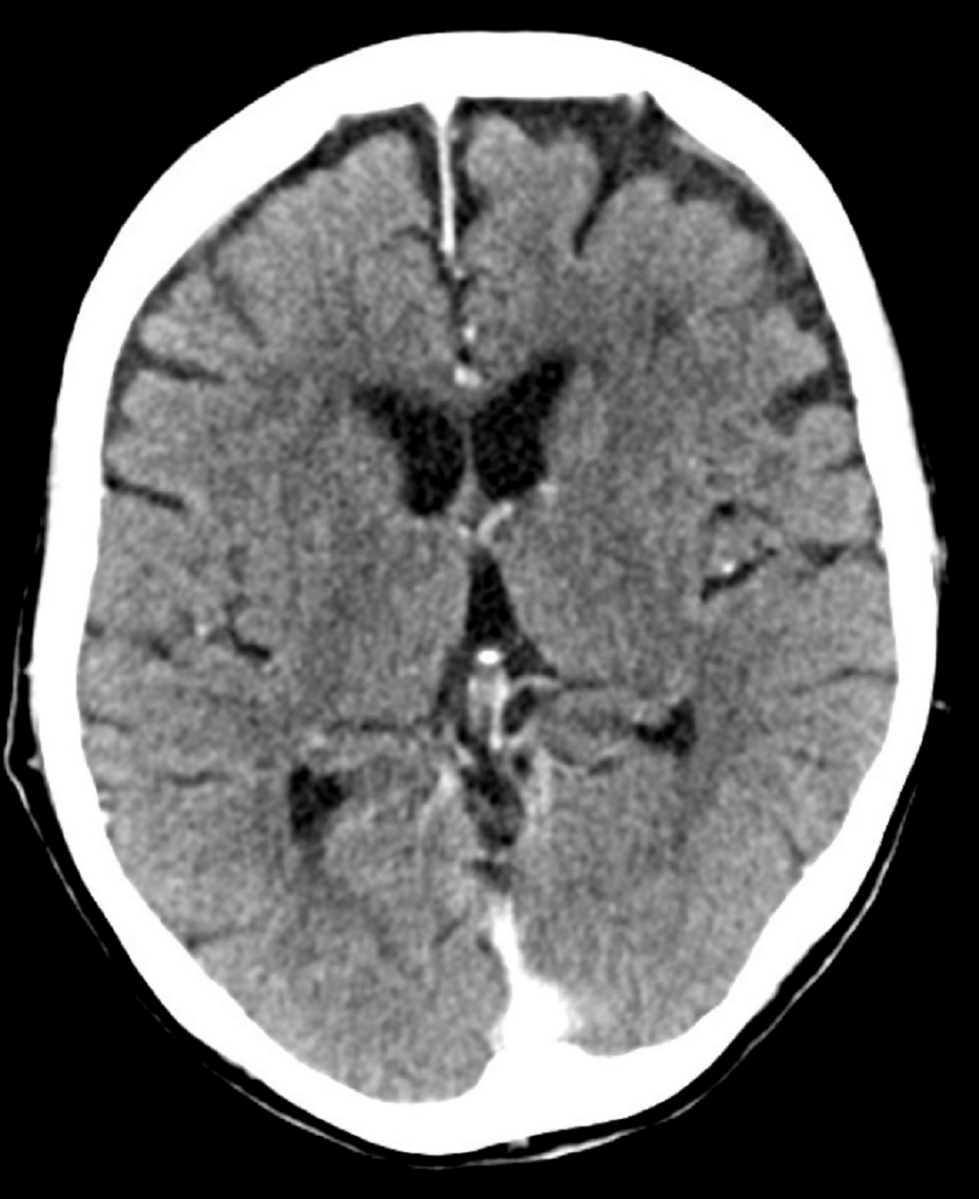
CT of the head with contrast showing no pathological intra- or extra-axial intracranial enhancement and no space-occupying lesion or surface collection CT: computed tomography

Given the persistently elevated inflammatory markers and ongoing confusion, she was empirically treated for meningoencephalitis with intravenous antibiotics pending further investigations. However, subsequent CSF and viral PCR results were unremarkable, effectively excluding infectious meningoencephalitis. CT scan of the head with contrast was normal, helping to exclude vascular, neoplastic, or structural lesions (Figure 1).

**Figure 2:**
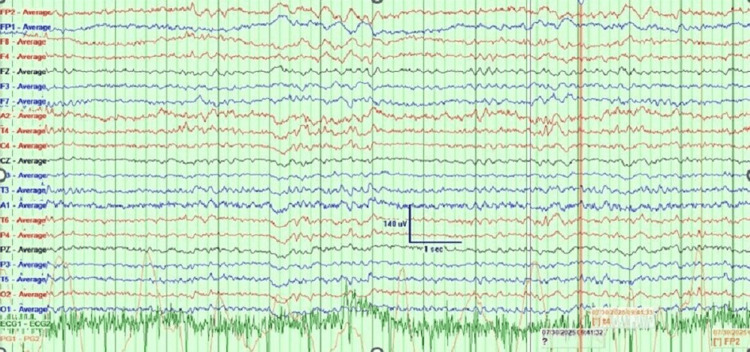
EEG recording showing captured low-amplitude suspicious spiky component over the right mid-temporal region, no definite epileptiform discharges, and no seizure activity EEG: electroencephalogram

Two electroencephalograms (EEGs) were performed during admission, two weeks apart, both showing non-specific focal cerebral dysfunction and low-amplitude spiky components without epileptiform discharges, showing no specific features for CJD. The initial EEG is presented in Figure 2.

**Figure 3:**
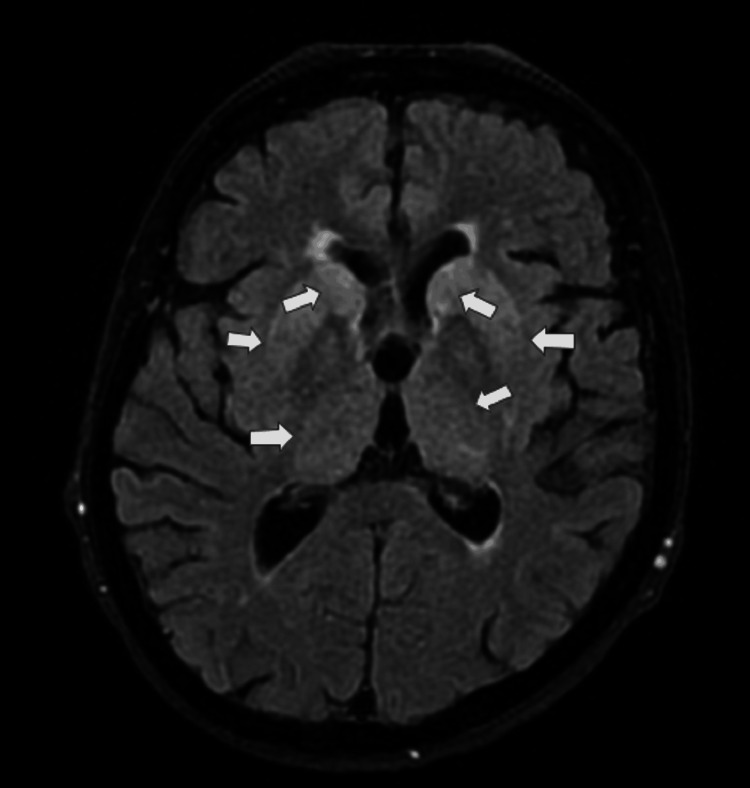
Axial FLAIR MRI showing bilateral hyperintense signal changes involving the caudate nuclei, lentiform nuclei, and thalami nuclei (white arrows). These deep grey matter abnormalities are suggestive of Creutzfeldt-Jakob disease FLAIR: fluid-attenuated inversion recovery; MRI: magnetic resonance imaging

**Figure 4:**
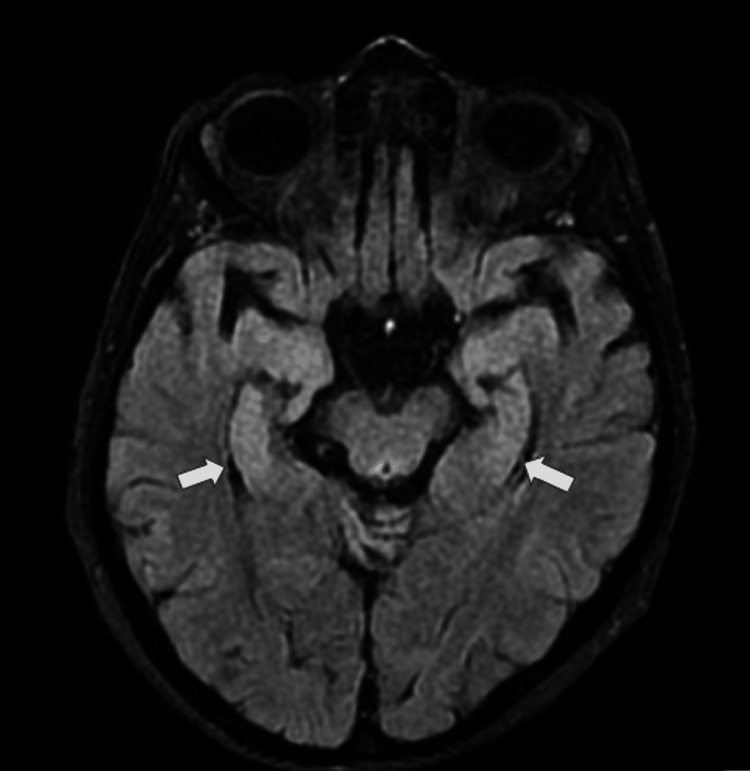
Axial FLAIR MRI showing bilateral hyperintense signal changes involving the hippocampi (while arrows). These grey matter abnormalities can be seen in Creutzfeldt-Jakob disease FLAIR: fluid-attenuated inversion recovery; MRI: magnetic resonance imaging

Due to uncertainty about the metalwork in the ear from remote surgery, there was a delay in obtaining magnetic resonance imaging (MRI); once it was confirmed MRI-compatible, MRI of the brain demonstrated abnormal fluid-attenuated inversion recovery (FLAIR) high signal associated with reduced diffusivity involving the bilateral caudate, ventral putamen, thalami (Figure 3), and hippocampi symmetrically (Figure 4). There were equivocal cortical signal changes involving the cingulate gyri. These appearances, in the context of her rapid decline, were highly suggestive of CJD. 

The combination of laboratory, CSF, and neurophysiological investigations prompted a comprehensive review of alternative diagnostic possibilities, with key differential diagnoses considered, such as autoimmune limbic encephalitis, metabolic conditions, and hypoxic-ischemic brain injury. The absence of a corresponding history made these conditions less likely and more in favor of CJD. 

The CSF sample for RT-QuIC was initially heavily blood-stained; therefore, a repeat test was requested. However, both the patient and her family declined further sampling, preferring to prioritize her comfort. Subsequently, she was referred to the National Creutzfeldt-Jakob Disease Research and Surveillance Unit in Edinburgh for specialized diagnostic testing. The team advised that RT-QuIC testing was not imperative for diagnosis, as the clinical presentation and MRI findings were already conclusive for probable sCJD. 

Despite supportive care, her clinical condition worsened rapidly to a bedbound state with marked cognitive decline. During admission, her speech became progressively softer with marked hypophonia. She also experienced intermittent episodes of mutism during the admission period, though she preserved her ability to swallow and was able to eat and drink, which provided her with comfort until she was discharged. Neurology confirmed that the picture was consistent with probable sCJD. The patient's condition continued to deteriorate and was looked after by the palliative care team who prescribed pro re nata (PRN) anticipatory medications and was discharged later to a hospice. She sadly passed away shortly after discharge. 

## Discussion

CJD is a rare, uniformly fatal prion disease characterized by rapidly progressive neuropsychiatric deterioration. This patient experienced a dramatic loss of independence that is characteristic of sCJD, with a rapid decline in her ability to perform everyday activities. After being previously fully active and independent, her functional status deteriorated within weeks, first becoming limited to sitting in a chair and ultimately becoming completely bedbound. Such a sharp decline in mobility and self-care over a brief period is typical in CJD, where progressive brain damage quickly leads to profound disability and total dependence [9]. The hallmark of sCJD is the rapidly advancing cognitive or functional decline often compounded by cerebellar ataxia, myoclonus, visual disturbances, and pyramidal or extrapyramidal features [10].

Our case highlights a rapidly progressive neurological decline in a previously active 73-year-old woman ultimately diagnosed with CJD. The case is notable for initial diagnostic uncertainty, overlapping symptoms with other neurological and paraneoplastic conditions, and the diagnostic challenges imposed by previous ear surgery that initially precluded MRI investigation.

Early recognition can be challenging due to the clinical overlap with more common neurological conditions such as Alzheimer's disease, vascular dementia, or rapidly progressive causes, including autoimmune encephalitis, viral encephalitis, paraneoplastic syndromes, and metabolic/toxic encephalopathies. In this case, a history of malignancy raised the possibility of paraneoplastic limbic or cerebellar syndromes, which added more complexity to the diagnostic process. It was documented that only 18% of patients with CJD were correctly diagnosed at first assessment [11].

Dizziness and vertigo are reported as initial manifestations, but occur in a minority of patients. Studies report that dizziness or vertigo as an onset symptom is present in about 20% of elderly sCJD cases [10]. For this patient, vertigo and cerebellar signs (nystagmus, dysmetria, and ataxia) were prominent and developed before the most severe cognitive decline, in keeping with CJD. The clinician should maintain a high index of suspicion for CJD in older adults developing new, rapidly progressive cerebellar signs and consider MRI in order to rule out reversible causes and not to delay the diagnosis.

Urinary incontinence was reported as one of the atypical symptoms of CJD, occurring in 13.3% of cases according to one cohort study of 15 patients conducted previously [8]. Although most patients with CJD develop urinary incontinence at a later stage in the disease course, in this case, it was noted at an early stage [7]. This symptom was attributed to pathological lesions in the prefrontal cortex and basal ganglia, as well as the sacral spinal cord, leading to the impairment of the micturition reflex [12].

MRI is the single most sensitive imaging modality for the diagnosis of CJD. Diffusion-weighted imaging (DWI) and FLAIR images were reported to have 91% sensitivity, 95% specificity, and 94% accuracy in differentiating CJD from other types of dementia [13]. In this particular case, MRI revealed bilateral hyperintensity changes in the basal ganglia, including the bilateral caudate, ventral putamen, thalamus, and hippocampi, findings that strongly supported the CJD diagnosis. Physicians should have a low threshold for MRI in patients presenting with progressive cognitive and functional decline.

It has been documented that periodic sharp wave complexes are observed on EEGs in about two-thirds of individuals diagnosed with sCJD. In the early stage of CJD, EEG can be non-specific, showing only diffuse slowing or non-descriptive findings [8]. Thus, a normal or non-diagnostic EEG should not delay further investigation especially with high clinical suspicion.

Probable diagnosis of sCJD can be supported by a combination of neuropsychiatric disorder and positive RT-QuIC assay performed on the CSF or other tissues [14]. The RT-QuIC assay on the CSF now offers 92-96% sensitivity and 100% specificity, exceeding the accuracy of prior "surrogate" markers (CSF 14-3-3 and total tau) [15]. In this process, a recombinant prion protein acts as a substrate, aggregating when pathogenic prion proteins are added. In this patient, the diagnosis of probable sCJD was established clinically and radiologically, despite the absence of RT-QuIC testing, reflecting the high diagnostic value of the characteristic history and MRI findings.

Management remains largely supportive, as no curative or disease-modifying therapy currently exists. Despite ongoing research, no pharmacologic agents have shown definitive efficacy in altering disease progression. Trials investigating quinacrine, flupirtine, and other agents have yielded inconclusive results [16,17]. The primary goals of care are to alleviate symptoms, optimize quality of life, and support caregivers during the disease course.

For symptom control, levetiracetam and benzodiazepines can be used for the management of troublesome myoclonus and other involuntary movements in CJD. Atypical antipsychotics seem to be of benefit for agitation, while cholinesterase inhibitors may be used to address hallucinations [18]. Multidisciplinary palliative care is essential and includes nutritional support, physical care to prevent complications such as aspiration pneumonia, and psychosocial support for both the patient and family. As the disease progresses, patients usually become akinetic and mute, requiring full-time care. Advanced care planning and early palliative involvement are therefore critical components of comprehensive management.

The prognosis of CJD is extremely poor. The mean survival of sCJD is four to eight months, with 90% of patients dying within a year [6]. Some case reports show a protracted course, e.g., one patient in Sri Lanka with sCJD had slow progression over two years [19].

Infection control in CJD remains critically important, although it is not transmitted by casual contact or conventional respiratory or droplet routes. This is due to the high resistance of prions to standard sterilization methods and the risk of iatrogenic transmission, particularly in surgical and procedural settings [20]. Standard precautions are typically sufficient for the routine care of CJD patients. However, enhanced precautions are required for invasive procedures involving high-risk tissues such as the brain, spinal cord, and eye. These include using single-use disposable instruments where possible and the incineration or enhanced sterilization of potentially contaminated instruments by high-temperature autoclaving following chemical treatment with sodium hydroxide or sodium hypochlorite [20].

## Conclusions

This case reinforces the importance of considering sCJD in elderly patients presenting with rapidly progressive neurological decline and cerebellar signs, even when some initial symptoms are atypical or confounded by comorbid conditions such as malignancy. Early urinary incontinence, as seen in this patient, is a rare initial manifestation in sCJD and may reflect the early involvement of the prefrontal and basal ganglia regions. Recognition of such uncommon features is important, as they can broaden the differential diagnosis and contribute to diagnostic delay. The case further highlights the diagnostic challenges posed by deferred MRI due to presumed contraindication and non-specific EEG findings, emphasizing the need to maintain a high index of suspicion for prion disease in patients with rapidly progressive dementia and cerebellar features. MRI remains the most sensitive imaging modality for sCJD, and timely imaging can guide appropriate confirmatory further testing. 

## References

[1] Norrito RL, Puleo MG, Pintus C (2024). Paraneoplastic cerebellar degeneration associated with breast cancer: a case report and a narrative review. Brain Sci.

[2] Katsikaki G, Dagklis IE, Angelopoulos P, Ntantos D, Prevezianou A, Bostantjopoulou S (2021). Atypical and early symptoms of sporadic Creutzfeldt - Jakob disease: case series and review of the literature. Int J Neurosci.

[3] Uttley L, Carroll C, Wong R, Hilton DA, Stevenson M (2020). Creutzfeldt-Jakob disease: a systematic review of global incidence, prevalence, infectivity, and incubation. Lancet Infect Dis.

[4] Kaur J, Lam MT, Singh S, Somal NK (2024). Slow to respond: a rapidly progressive case of sporadic Creutzfeldt-Jakob disease. Cureus.

[5] Manara R, Fragiacomo F, Ladogana A (2024). MRI abnormalities in Creutzfeldt-Jakob disease and other rapidly progressive dementia. J Neurol.

[6] Hall WA, Masood W (2025). Creutzfeldt-Jakob disease. StatPearls [Internet].

[7] (2025). Symptomatic treatment. https://www.ucl.ac.uk/national-prion-clinic/symptomatic-treatment.

[8] Liao J, Hu W, Chen S (2024). Multidimensional features of sporadic Creutzfeldt-Jakob disease in the elderly: a case report and systematic review. Front Aging Neurosci.

[9] Kojima G, Tatsuno BK, Inaba M, Velligas S, Masaki K, Liow KK (2013). Creutzfeldt-Jakob disease: a case report and differential diagnoses. Hawaii J Med Public Health.

[10] Manix M, Kalakoti P, Henry M, Thakur J, Menger R, Guthikonda B, Nanda A (2015). Creutzfeldt-Jakob disease: updated diagnostic criteria, treatment algorithm, and the utility of brain biopsy. Neurosurg Focus.

[11] Paterson RW, Torres-Chae CC, Kuo AL (2012). Differential diagnosis of Jakob-Creutzfeldt disease. Arch Neurol.

[12] Yano M, Sakakibara R, Tateno F (2016). Urodynamic findings in patients with Creutzfeldt-Jakob disease: a case report. Int Urol Nephrol.

[13] Young GS, Geschwind MD, Fischbein NJ (2005). Diffusion-weighted and fluid-attenuated inversion recovery imaging in Creutzfeldt-Jakob disease: high sensitivity and specificity for diagnosis. AJNR Am J Neuroradiol.

[14] (2025). CJD diagnostic criteria. https://www.cdc.gov/creutzfeldt-jakob/hcp/clinical-overview/diagnosis.html.

[15] Fiorini M, Iselle G, Perra D (2020). High diagnostic accuracy of RT-QuIC assay in a prospective study of patients with suspected sCJD. Int J Mol Sci.

[16] Haïk S, Brandel JP, Salomon D (2004). Compassionate use of quinacrine in Creutzfeldt-Jakob disease fails to show significant effects. Neurology.

[17] Otto M, Cepek L, Ratzka P (2004). Efficacy of flupirtine on cognitive function in patients with CJD: a double-blind study. Neurology.

[18] John C, Johnny T, Hatice K (2023). A systematic review of pharmacological treatments for motor symptoms in Creutzfeldt Jakob disease (CJD). J Neurol Neurosurg Psychiatry.

[19] Subhani B, Rajaratnam A, Senanayake B (2025). An unusually protracted case of sporadic Creutzfeldt-Jakob disease highlighting diagnostic challenges in a low-resource setting: a case report. J Cey Coll Phy.

[20] (2025). Infection control for CJD. https://www.cdc.gov/creutzfeldt-jakob/hcp/infection-control/index.html.

